# Visualization analysis of research hotspots on structural topology optimization based on CiteSpace

**DOI:** 10.1038/s41598-023-45447-y

**Published:** 2023-10-24

**Authors:** Yi Zhong, Xue-tao Jiang, Yong Yang, Ben-lian Xu, Qi-xin Zhu, Lei Wang, Xin-feng Dong

**Affiliations:** 1 College of Mechanical Engineering, Suzhou University of Science and Technology, Suzhou, People’s Republic of China; 2 School of Electronic & Information Engineering, Suzhou University of Science and Technology, Suzhou, People’s Republic of China; 3 School of Electromechanical and Vehicle Engineering, Weifang University, Weifang, People’s Republic of China

**Keywords:** Engineering, Materials science

## Abstract

Structural topology optimization has gained widespread attention due to more possibilities of innovative structural design. The current research focus/hotspots, application areas, main research scholars, institutions and the countries involved in structural topology optimization are visually presented through clustering and visual analysis based on CiteSpace. The four metric dimensions of the literatures in this paper are as follows: annual quantity of papers and core countries, core authors and co-authors’ institutions, hotspots and burst terms, and the highly co-cited papers. The results show the research hotspots in this field are concentrated on keywords such as "level set method", "sensitivity analysis", "homogenization", "genetic algorithm", etc. Regarding the research frontier, “moving morphable component (MMC)”, “additive manufacturing (AM)” and “deep learning” are hot topics. In addition, Y. Sui, Z. Kang and O. Sigmund, etc. have high publications. M. Bendsøe and O. Sigmund have high citations. Dalian University of Technology, Technical University of Denmark, etc. are prominent institutions. Moreover, China accounts for more than 34% in the terms of original WOS literatures following by the USA and Australia. This paper could identify structural topology optimization development patterns for the scholars concerned with this field, especially novices, to quickly focus and track the research priorities.

## Introduction

Structural optimization design is a new technology that has developed over the past forty years. The design goals and the required constraints are organically combined in structural optimization design. The optimal structure in the feasible domain of the design constraint can be realized by optimization theory and method, so as to obtain the best design scheme. Structural optimization design includes size optimization, shape optimization, and topology optimization. The size optimization and shape optimization technologies are currently well-developed. However, once the structural layout is determined, the potential for design modifications becomes limited, thereby restricting the effectiveness of optimization designs. Structural topology optimization aims to determine the optimal distribution of structural materials based on constraints, loads. From a macroscopic perspective, topology optimization encompasses not only the section and geometry of the structure but also its composition in terms of topology models, i.e., the spatial connectivity mode of its components. Due to its more possibilities of innovative structural design, topology optimization has been widely applied in the fields of engineering manufacturing and infrastructure construction fields, etc.

By iteratively calculating according to the optimization criteria, the optimal topology shape for the certain performance could be obtained. Structural topology optimization offers significant advantages in the following aspects. Firstly, it enables weight reduction and enhances the strength-to-mass ratio of structures, thereby reducing material costs and energy consumption. Secondly, it mitigates stress concentration phenomena within structures, leading to improved fatigue life and enhanced safety. Thirdly, structural topology optimization minimizes processing steps and material waste in manufacturing processes, resulting in reduced production costs and shorter cycles. Lastly, structural topology optimization can improve the dynamic performance of the structure, change the natural frequency and modal form of the structure, and reduce the vibration response and noise radiation of the structure. Consequently, structural topology optimization has found successful applications across various disciplines such as construction, chemical industry, metallurgy, automatic control systems, fluid flow, dynamics, acoustics, and wave propagation, aerospace design and aeroelasticity, biomedical design, multifunctional materials and other fields.

Research on structural topology optimization of discrete structures can be traced back to the early twentieth century. A. Michell et al.^1^ adopted the analytical method to realize the optimal design of two-bar truss structure under the constraint of single loading stress. This classic achievement is Michell theory. However, this theory could only be applied in the single working condition. Moreover, there are high requirements on strain field. Until the early 1960s, W. Dorn, R. Gomory et al.^2^ introduced ground structure method, combined numerical methods. The study conducted by D. Goldberg^3^ marked the pioneering application of genetic algorithms (GA) in the field of structural optimization. Based on the concept of independent continuous topological variables and the idea of mapping change, Y. Sui et al.^4^ proposed Independent Continuous Mapping (ICM) that can realize the transformation between discrete variables and continuous variables. During the development of continuous structure topology optimization, in the early 1980s, K. Cheng et al.^5^ innovatively pointed out that the optimal distribution of solid elastic plates is composed of numerous small dense rib stiffeners. This achievement inspired M. Bendsøe and N. Kikuchi^6^, who proposed the homogenization method. Y. Xie^7^ proposed evolutionary structure optimization (ESO) in 1992. O. Querin develops ESO into a bidirectional evolutionary algorithm BESO^8^. Starting from a simple design area, Querin effectively removes or adds elements in the iterative process according to certain criteria to achieve self-growth of the structure. V. Young^9^ applied BESO to multi-condition situations and solved 2D and 3D problems. The topology optimization problem of macroscopic model is described by the size of material microstructure in this method. After the emergence of the finite element method, the solid isotropic material with penalization (SIMP) method is proposed by M. Bendsøe and O. Sigmund^10^. SIMP could improve the computational efficiency of structural topology optimization. In 1988, S. OSHER and J. SETHIAN^11^ proposed an approach called the level-set method. This method describes the boundary of the structure by introducing the isosurface of the level set function. In addition, the specific velocity field is set, and the boundary condition of the structure at the next moment is obtained by solving Hamilton–Jacobi equation in this method. In 2014, Professor X. Guo et al.^12,13^ from Dalian University of Technology proposed the moving morphable component (MMC) approach. The non-gradient optimization method based on MMC parameterization can overcome the problem that gradient information is difficult to obtain or unreliable. It can optimize structure topology without relying on gradient information. The evolutionary level set method is proposed aiming to minimize the peak acceleration and maximize the energy absorption capacity to optimize the topology of the structure^14^. Evolutionary Algorithms (EAs) together with a suitable low-dimensional representation in an extended version of the Evolutionary Level Set Method (EA-LSM) is proposed to find the optimal design that minimizes the peak stress and maximizes the energy absorption capacity of the joints under crash loads^15^. The nine non-gradient approach based approaches based on the moving morphable are compared by the convergence speed, the quality of fnal designs, and the abilities to explore and exploit based on a diversity index^16^. An evolutionary algorithm based on coevolution and cooperation is proposed using a divide-and-conquer strategy to improve the efficiency and convergence speed of evolutionary topology optimization based on MMC^17^. W. Zhang et al.^18^ proposed the moving morphable void method (MMV) approach.

The traditional topology optimization method depending on gradient has some limitations when the inability to consider complex collision loads and multi-physics coupling or there is numerical noise in the gradient information in unconventional cases such as crashworthiness design. In recent years, the research and development of cellular automata (CA) has created a new direction to solve this design problem. The Hybrid Cellular Automata (HCA) combines CA with finite element analysis. HCA method and its improved method modified hybrid cellular automata (MHCA) have great potential in the optimization of collision avoidance performance in the automotive field^19,20^, and can provide a more efficient and reliable design scheme. At the same time, the improved algorithm based on HCA is suitable for special structural optimization problems, such as multiscale topology optimization for non-uniform microstructure^21^, topology optimization design of three‑dimensional multi‑material and multi‑body structure^22^. The new hybrid methods for the identification of optimal topologies by combining the teaching–learning based optimization (TLBO) and the method of moving asymptotes (MMA) for handling the numerical noise in topology optimization is presented^23^. In order to find cross-section designs of crashworthiness profiles, a novel version of the Graph and Heuristic based Topology Optimization (GHT) was proposed via the combination of mathematical optimization algorithms with heuristics that are based on expert knowledge^24^.

With the increasing complexity of engineering design, multi-objective optimization technology has gradually become a research hotspot in the field of structural topology optimization. The aim of structural topology optimization is to find the optimal shape of a structure to meet multiple objectives, such as minimum weight, maximum stiffness and minimum stress. These objectives are often contradictory, so multi-objective optimization is required to balance the tradeoffs between them. The topology optimization problem is formulated as a multi-objective optimization problem by simultaneously considering objectives of coverage, propagation intensity and interference intensity as well as the constraint of connectivity, to address the challenges in designing efficient and reliable wireless data center networks^25^. A multi-objective optimization framework is proposed using random optimization strategy to solve the challenge of dense reconstruction under fast camera motion. ROSEFusion outperforms existing methods in terms of both accuracy and efficiency^26^. Multi-objective graph-based differential grouping with shift (mogDG-shift) to decompose the large number of variables in an MOLSOP is proposed to solve multi-objective large-scale optimization problems^27^. The multi-objective optimization problem is very common in the optimization of power system^28,29^.

With the rapid development of computing technology and the increasing maturity of artificial intelligence technology, machine learning algorithms represented by deep learning have achieved unprecedented development. This provides more possibilities for shorter computation time and better results in structural topology optimization. For example, X. Lei et al.^30^ established a machine learning model based on the MMC topology optimization framework and provided preliminary predictions of the topology configuration. This significantly reduced the computational cost while meeting the accuracy requirements. I. Sosnovik et al.^31^ combined deep learning models with the SIMP method. They used a lightweight convolutional neural networks (CNN) model^32^ to simulate the process of topology optimization prediction. In this research, by using the initial information of the preliminary iterative structural density distribution and gradient values, reliable topology configurations could be easily obtained. In recent years, an increasing number of scholars have been using artificial intelligence technology to solve complex structural topology optimization problems.

The development process, current research hotspots and future research trends in the field of structural topology optimization are visually presented through clustering and visual analysis based on CiteSpace. The metric analysis of the documents includes four parts: annual quantity of papers and productive countries, core authors and co-authors’ institutions, hotspots and burst terms, and the highly co-cited papers. By combing the existing research in this field, the foundation for the formation of a mature interdisciplinary and multi-branch research system of structural topology optimization could be provided.

## Definition, objective and the main method of structural topology optimization

### Definition and objective

The objective of structural topology optimization is to satisfy constraint conditions such as stress and displacement by optimizing the allocation of material and quantity in the design domain, material layout and node connection mode, as well as transfer the load to the support position of the structure, so as to seek the optimal performance index of the structure. The objective function in topology optimization problems typically encompasses the stiffness, volume, cost, natural frequency, and amplitude of the structure. According to M. Bendsøe's optimal distribution model of materials^6^, the problem of structural topology optimization can be defined as spatial allocation of materials within the design space^33^.

1 $$x\left( x \right)\left\{ {\begin{array}{*{20}c} 1 & {if\,x \in \Omega _{s} } \\ 0 & {if\,x \in \Omega /\Omega _{s} } \\ \end{array} } \right.$$

**Figure 1:**
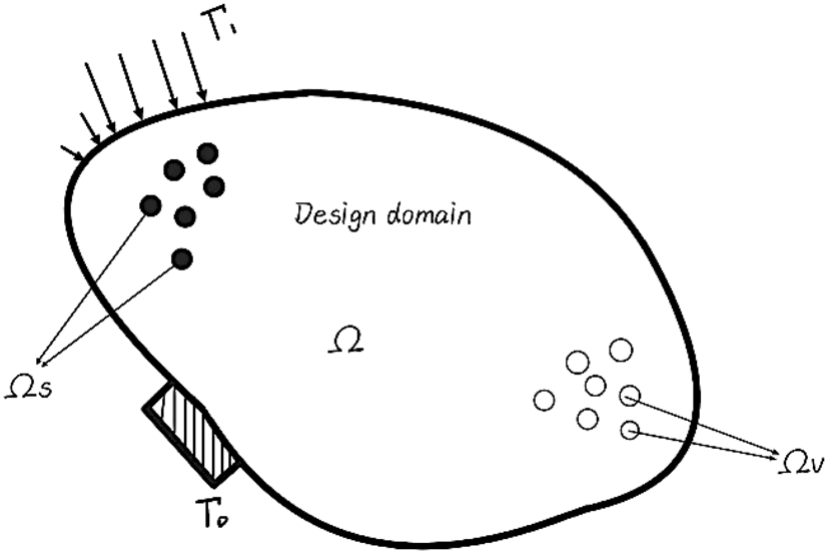
Design Domain and its Boundary Conditions.

In Eq. (1), $\Omega$ denotes given design area, ${\Omega }_{s}$ denotes the area occupied by the physical material, $\Omega /{\Omega }_{s}$ denotes the area occupied by the hole. The model of the design domain and its boundary conditions is shown in Fig. 1.

Topological optimization problems can be mathematically described as the following equations^34^:

2 $$\begin{array}{*{20}l} {\min } \hfill & {f_{Obj} \left( x \right)} \hfill \\ {s.t} \hfill & {g_{j} \left( x \right) \le 0} \hfill \\ {} \hfill & {h_{k} (x) = 0} \hfill \\ {} \hfill & {x_{\min } \le x_{i} \le x_{\max } } \hfill \\ {} \hfill & {i = 1,2, \cdot \cdot \cdot ,n_{x} } \hfill \\ {} \hfill & {j = 1,2, \cdot \cdot \cdot ,n_{g} } \hfill \\ {} \hfill & {k = 1,2, \cdot \cdot \cdot ,n_{h} } \hfill \\ \end{array}$$

In Eq. (2), ${f}_{Obj}$ denotes optimization objective function, $x$ denotes optimization variable array, $g$ denotes the inequality constraint, $h$ denotes the equation constraints, ${n}_{x}$ denotes the number of optimization variables, ${n}_{g}$ denotes the number of inequality constraints, ${n}_{h}$ denotes the number of equality constraints, ${x}_{min}$ and ${x}_{max}$ denotes the minimum and maximum values of the variable.

### The main methods and category

In structural topology optimization, common methods can be divided into two categories according to whether they are gradient-based or non-gradient.

Method of Moving Asymptotes (MMA), Optimality Criteria Method (OC), Solid Isotropic Material Penalization (SIMP), etc. optimize the structure shape by iteratively adjusting design variables and constraints. These methods use gradient information to realize topology optimization.

Evolutionary Structural Optimization (ESO) realizes optimizing structural form by simulating the process of growth and survival of the fittest. The optimal solution is searched by simulating the genetic and natural selection mechanisms in the process of biological evolution in Genetic Algorithm (GA). Hybrid Cellular Automaton (HCA) combines cellular automata and finite element method, updating by iteration alternately. Topology optimization is achieved by moving and deforming the basic components of the structure in Moving Morphable Components (MMC). Topology optimization is achieved by moving and deforming the distribution of voids in Moving Morphologies of Void (MMV).

Gradient-based methods usually have fast convergence speed and local search ability, but there are some limitations in global search and robustness. Non-gradient methods have the advantages of global search and robustness, but may be inferior in convergence speed and local search ability. How to choose the suitable method depends on the specific problem and requirements.

## Data sources, search strategy and research tool

### Data sources and search strategy

The publications are sourced from the China National Knowledge Infrastructure (CNKI) and the Web of Science Core Collection (WOSCC). The strategy used during the search is Topic search #1 = (“structural topology optimization” and then, the results are refined by [Timespan: 1999-01-01 to 2022-12-31]. A total of 4810 CNKI articles and 6221 WOS articles are obtained.

In order to enhance the value of this analysis, the result of CNKI literature is refined by [Journal: SCI, EI, PKU, CSSCI, CSCD, and AMI] to eliminate conference articles, newspaper articles, etc. After data cleaning, duplicate publications are removed by CiteSpace v.6.2.R2 in WOS literature; and ultimately, a total of 1631 CNKI and 6204 WOS unique records are used in the final analysis.

### Research tool

CiteSpace v.6.2. R 2 is a Java application used for analyzing and visualizing co-citation networks^35^. The software was developed by Professor Chaomei Chen's team at Drexel University in the United States. New trends and dynamics in the development of scientific documents could be identified and visualized based on CiteSpace. The relationships between scientific documents could be visually displayed to users through scientific knowledge maps. Moreover, CiteSpace can clearly sort out the track of past research and intuitively display the current research hotspots and future research prospects in the field^36^.

Firstly, the input literature data is processed through CiteSpace, including removing duplicate references, extracting keywords and reference relationships. Then, CiteSpace standardized the construction of the co-occurrence matrix of the processed data through COSINE, PIM, DICE and JACCARD. Further, the co-occurrence matrix is transformed into a visual co-occurrence graph through the layout algorithm on CiteSpace. Finally, the co-occurrence map is visualized based on the layout results by CiteSpace.

During the data analysis process based on CiteSpace, the time range is set from 1999 to 2022. “Pathfinder” and “Pruning sliced networks” are selected to simplify the view. Then, according to the requirements of the analysis, "Author", "Institution", "Country", "Keyword" “Category” and “Cited Author” are selected as clustering options to obtain co-occurrence maps or clustering maps of authors, institutions, countries, keywords, category and cited author. The research parameters are shown in Table 1.

**Table 1:** Parameters and their values for bibliometric analysis of structural topology optimization.

Parameters	Value
Year duration	1999–2022
Term source	Title, Abstract, Author Keywords and Keywords Plus
Node type	Author, Institution, Country, Keyword, Cited Author, Category
Pruning	Pruning and pathfinder sliced network
Links	Defaulting
Visualization	Cluster view static and show the merged network

## Analysis of the quantity, country and application areas of literature

### Analysis of the quantity and country of literature

The number of publications on the theme of structural topology optimization in the CNKI documents has been increasing year by year since 1999. There was a significant increase from 2003 to 2008, with the number of annual publications increasing from 6 in 1999 to 80 in 2008. From 2008 to 2018, the number of annual publications fluctuated slightly but continued to grow slowly. It reached a peak of 122 publications in 2022. Overall, the average number of annual publications from 1999 to 2022 was 68.

**Figure 2:**
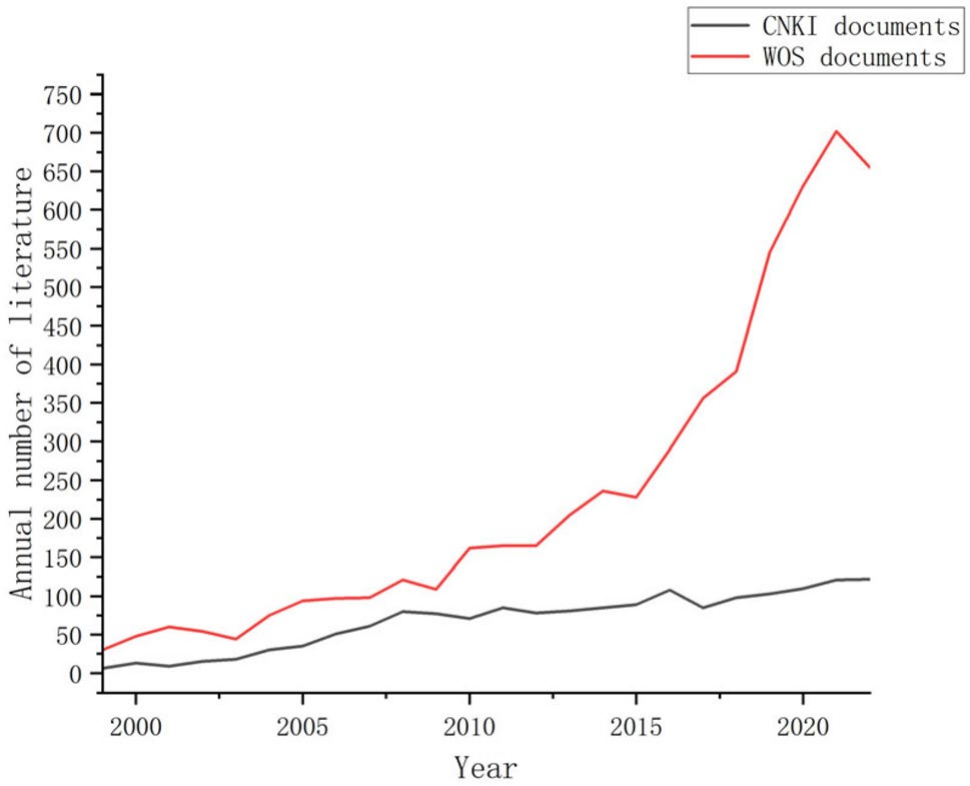
Annual number of literature published.

In the WOS documents with structural topology optimization as the theme, the overall number of annual publications has shown a year-on-year increase from 1999 to 2022. The average number of annual publications is 232 articles. In 1999, there were only 30 articles related to this field. The number of annual publications slowly increased until it reached 228 articles in 2015. From 2016 to 2021, with the development of science and technology, structural topology optimization has also received strong development. The number of annual publications has grown rapidly since then, and it reached 702 articles in 2021. As shown in Fig. 2, the overall trend of the number of annual publications of the CNKI and WOS documents in the field of structural topology optimization is almost the same, showing a fluctuating upward trend. However, in this field, the number of the WOS literature and the growth trend of annual publications far exceed those of the CNKI literature, indicating that the amount of the CNKI literature in this field is relatively small.

**Figure 3:**
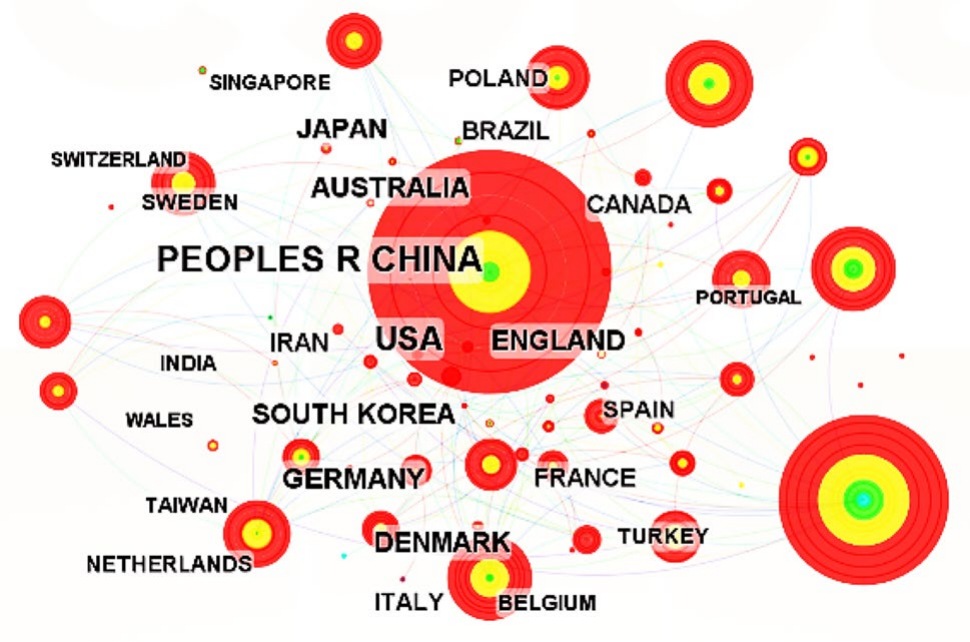
CS map of country co-authorship study in the WOS documents of structural topology optimization.

In CiteSpace, the time range from 1999 to 2022 is set with a time slice of 8 years and "country" is selected as the clustering option to obtain a CS map of country co-authorship study in structural topology optimization, which is shown in Fig. 3.

Through statistical analysis of information on the Fig. 3, Table 2 depicts the top ten fruitful countries ranked by the quantity of their published original articles in descendant order. China, the United States, and Australia ranked as the top three countries in terms of publication volume. China contributed 2129 papers, following the USA (1149), and Australia (417). The number of literatures from China accounts for 34.30% of the total amount of the WOS documents, far exceeding that of other countries. Although the quantity of publications in the CNKI is lower than that of the WOS publications, the proportion of Chinese original published papers in the WOS papers is far more than that of other country. This shows that Chinese scholars in the field of structural topology optimization have made undeniable contributions to some extent.

**Table 2:** Top 10 productive countries in WOS literature of structural topology optimization research extracted through CS analysis.

Name of country	Count	Year
PEOPLES R CHINA	2129	1999
USA	1149	1999
AUSTRALIA	417	1999
SOUTH KOREA	403	1999
GERMANY	388	1999
ENGLAND	308	1999
JAPAN	282	1999
DENMARK	264	1999
BRAZIL	235	1999
FRANCE	213	2001

### Analysis of the application areas

In order to make readers understand the application fields of structural topology optimization, statistical analysis of the application fields in structural topology optimization in WOS literatures is conducted with "Category" as the node selection criterion in CiteSpace. The analysis parameters and result parameters are shown in Table 3.

**Table 3:** Analysis parameters and result parameters for application areas analysis in WOS literatures.

	Parameters	Value
Analysis	Years per slice	11 years
	Node type	Category
	Selection criteria	Top 40%
	Pruning	None
Result	Nodes	55
	Links	233
	Density	0.1569

According to the analysis result of the WOS literature data obtained through the search strategy used in this paper, the application fields of structural topology optimization include the following four broad categories: engineering, biochemistry, computer and other interdisciplinary field.

**Figure 4:**
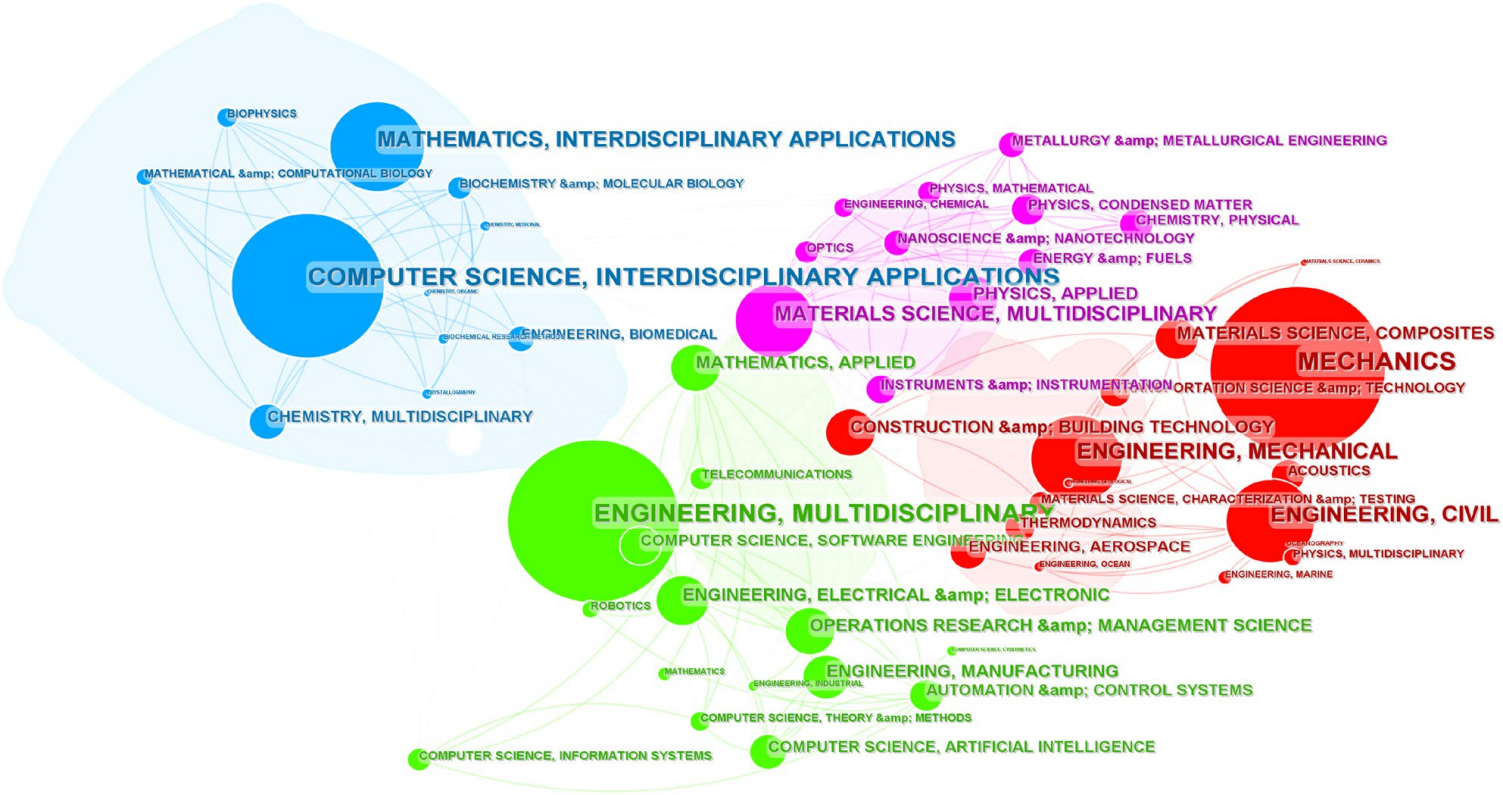
CS map of application areas analysis in the WOS literatures.

The first category, engineering fields include: machinery, civil engineering, manufacturing, aerospace, materials, transportation, Marine, environment, etc. The second category, pharmaceutical chemistry, biochemical research, biophysics and so on. The third category, software engineering, artificial intelligence, information systems, cybernetics, etc. The CS map of application areas analysis in the WOS literatures is shown in Fig. 4.

In the Table 4, the top five application areas are listed according to the "count" indicator.

**Table 4:** Top 5 application areas of structural topology optimization in WOS literatures.

Categories	Count	Centrality
Mechanics	2827	0.16
Engineering, multidisciplinary	2805	0.09
Computer science, interdisciplinary applications	2131	0.43
Mathematics, interdisciplinary applications	822	0.01
Engineering, mechanical	765	0.19

## Author, co-cited author and co-authors’ institutions analysis

### Author co-authorship analysis

In CiteSpace, statistical analysis of core scholars in structural topology optimization is conducted. It is worth noting that the number of published articles is only a small reference indicator of the scientific research level of scholars, not a decisive factor."

**Figure 5:**
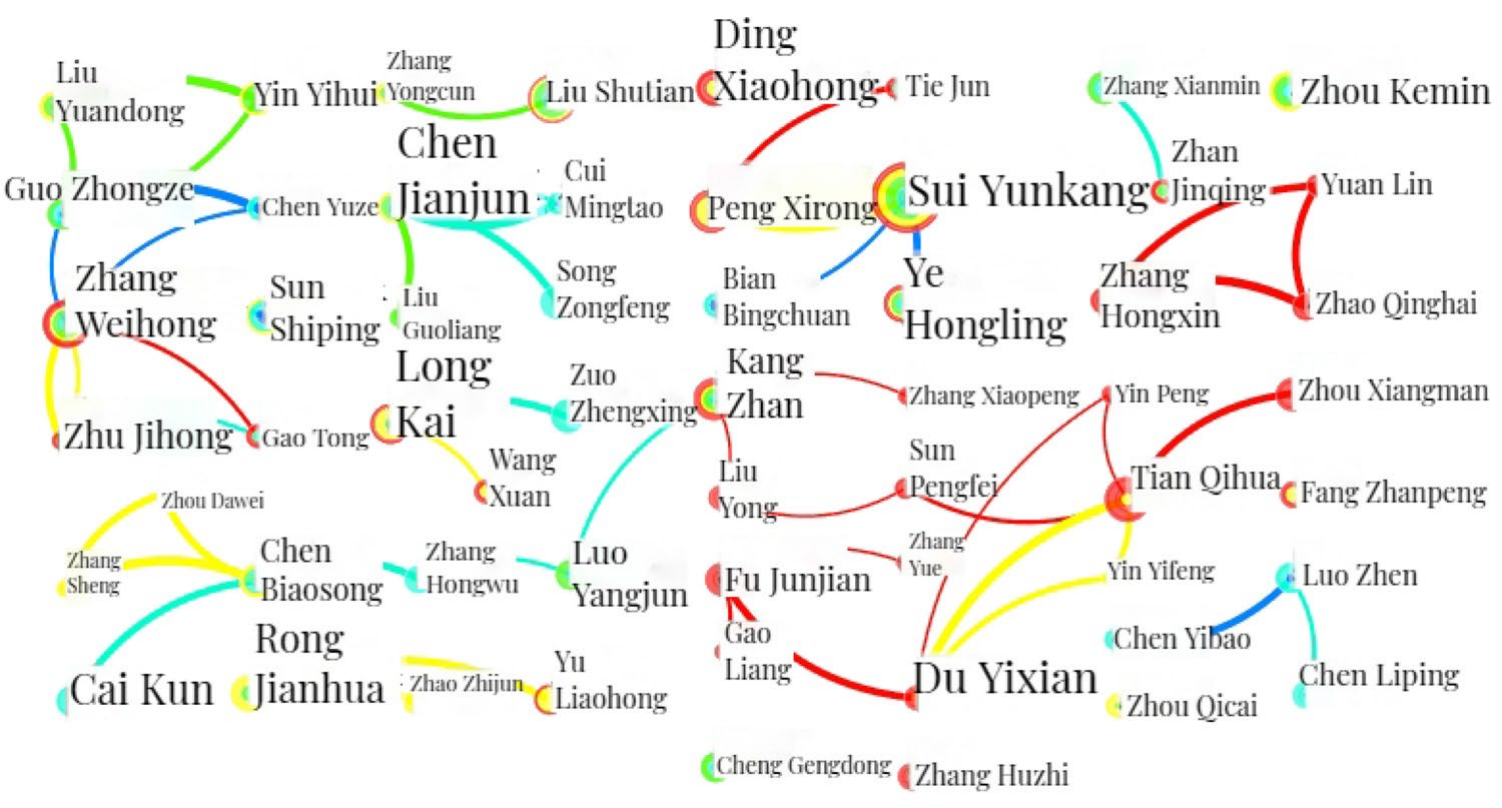
CS map of author co-authorship analysis in the CNKI documents.

A time slice of 4 years is set for the CNKI documents, and the top 50 data are selected. "Author" is set as the node type. Additionally, the view is simplified by selecting “Pathfinder” and “Pruning sliced networks”, resulting in a CS map of author co-authorship analysis in the CNKI documents which is shown in Fig. 5. Figure 5 comprises 320 nodes and 230 links, having a density of 0.0045. The analysis parameters and result parameters are shown in Table 5.

**Table 5:** Analysis parameters and result parameters for author co-authorship analysis in CNKI literatures.

	Parameters	Value
Analysis	Years per slice	4 years
	Node type	Author
	Selection criteria	Top 50%
	Pruning	Pruning and pathfinder sliced network
Result	Nodes	320
	Links	230
	Density	0.0045

In the CS map of author co-authorship, nodes represent the authors of published papers. The node size denotes the total number of articles published by authors. That is to say the more papers an author published, the larger the node is. Conversely, the smaller their node is. Meanwhile, the thickness of the links shows that the authors cooperate. The thicker the link is, the stronger the collaborative relationship between scholars is.

As shown in Fig. 5, Y. Sui^37^, W. Zhang^38^, H. Ye^39^, X. Ding^40^, K. Long^41^, X. Peng^42^, J. Rong^43^ and J. Chen^44^ have published more papers. Based on the analysis, the top ten scholars with high publications in the field of structural topology optimization of the CNKI documents was sorted by the total number of published articles, as shown in Table 6.

**Table 6:** Top 10 scholars with high publications in the field of structural topology optimization in the CNKI literatures.

Name of author	Count	Year
Y. Sui	58	2005
W. Zhang	37	2006
H. Ye	30	2005
X. Ding	29	2008
K. Long	27	2007
J. Rong	25	2005
X. Peng	25	2006
J. Chen	20	2006
J. Zhu	19	2008
Y. Du	18	2011

Y. Sui ranks first in high publication in the field of structural topology optimization, with 58 papers published. W. Zhang has published 37 articles, followed by H. Ye (30), X. Ding (29), K. Long (27), X. Peng (25), J. Rong (25), and J. Chen (20). Other authors have published less than 20 articles each.

**Figure 6:**
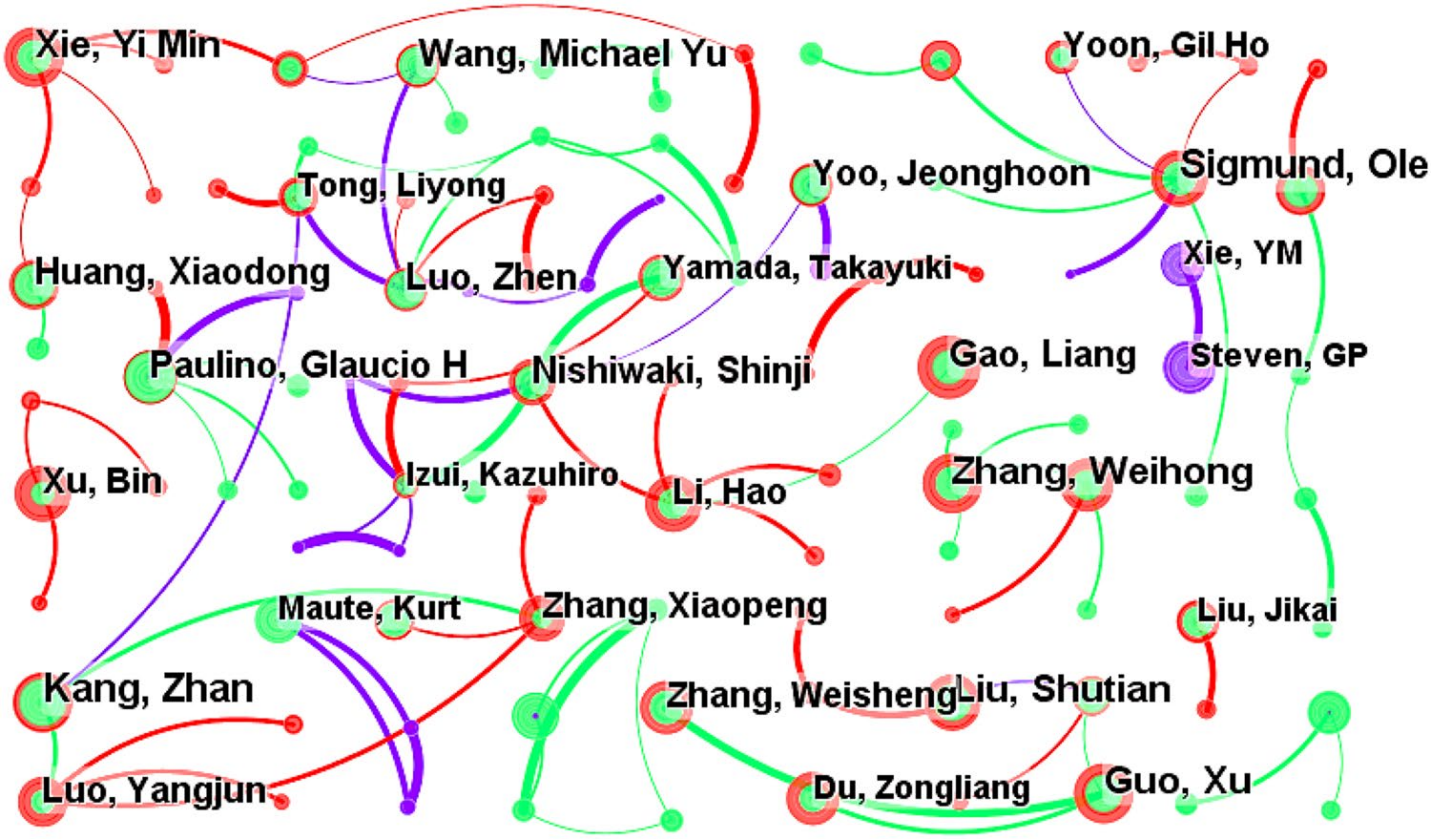
CS map of author co-authorship analysis in the WOS documents.

The WOS documents are analyzed with a time slice of 11 years and the top 50 data are selected. The node type is set as "Author". The “Pathfinder” and “Pruning sliced networks” are selected to simplify the view. Figure 6 shows the CS map of author co-authorship analysis in the WOS documents. There were 475 nodes and 509 links, having a density of 0.0045. The analysis parameters and result parameters are shown in Table 7.

**Table 7:** Analysis parameters and result parameters for author co-authorship analysis in WOS literatures.

	Parameters	Value
Analysis	Years per slice	11 years
	Node type	Author
	Selection criteria	Top 50%
	Pruning	Pruning and pathfinder sliced network
Result	Nodes	475
	Links	509
	Density	0.0045

As shown in Fig. 6, Z. Kang^45^, O. Sigmund^46^, W. Zhang^47^, W. Zhang^48^ are authors with relatively bigger node, indicating that they have published more papers. Through analysis, the information of the top ten scholars with high publication in the field of structural topology optimization in the WOS literatures was obtained, which is shown in Table 8.

**Table 8:** Top 10 scholars with high publication in the field of structural topology optimization in the WOS literatures.

Name of author	Count	Year
Z. Kang	86	2008
O. Sigmund	86	2006
W. Zhang	71	2006
Y. Xie	70	2010
X. Guo	66	2010
S. Liu	65	2008
G. Paulino	60	2009
X. Huang	57	2010
M. Wang	56	2006
S. Nishiwaki	56	2006

Z. Kang and O. Sigmund ranked first with 86 papers. W. Zhang and Y. Xie^49^ also published more than 70 papers, while other scholars published 55 to 70 papers. It shows that among the top ten scholars with high publication in the WOS literatures in this field, there are 7 Chinese authors. The proportion of Chinese authors is 70%. This is far more than the proportion of other countries.

### Cited author analysis

In order to let readers have more indicators to understand the scholars in the field of structural topology optimization, the cited author is analyzed. The analysis parameters and result parameters are shown in Table 9.

**Table 9:** Analysis parameters and result parameters for cited author analysis in the WOS literatures.

	Parameters	Value
Analysis	Years per slice	11 years
	Node type	Cited author
	Selection criteria	Top 40%
	Pruning	Pruning and pathfinder sliced network
Result	Nodes	78
	Links	85
	Density	0.0283

**Figure 7:**
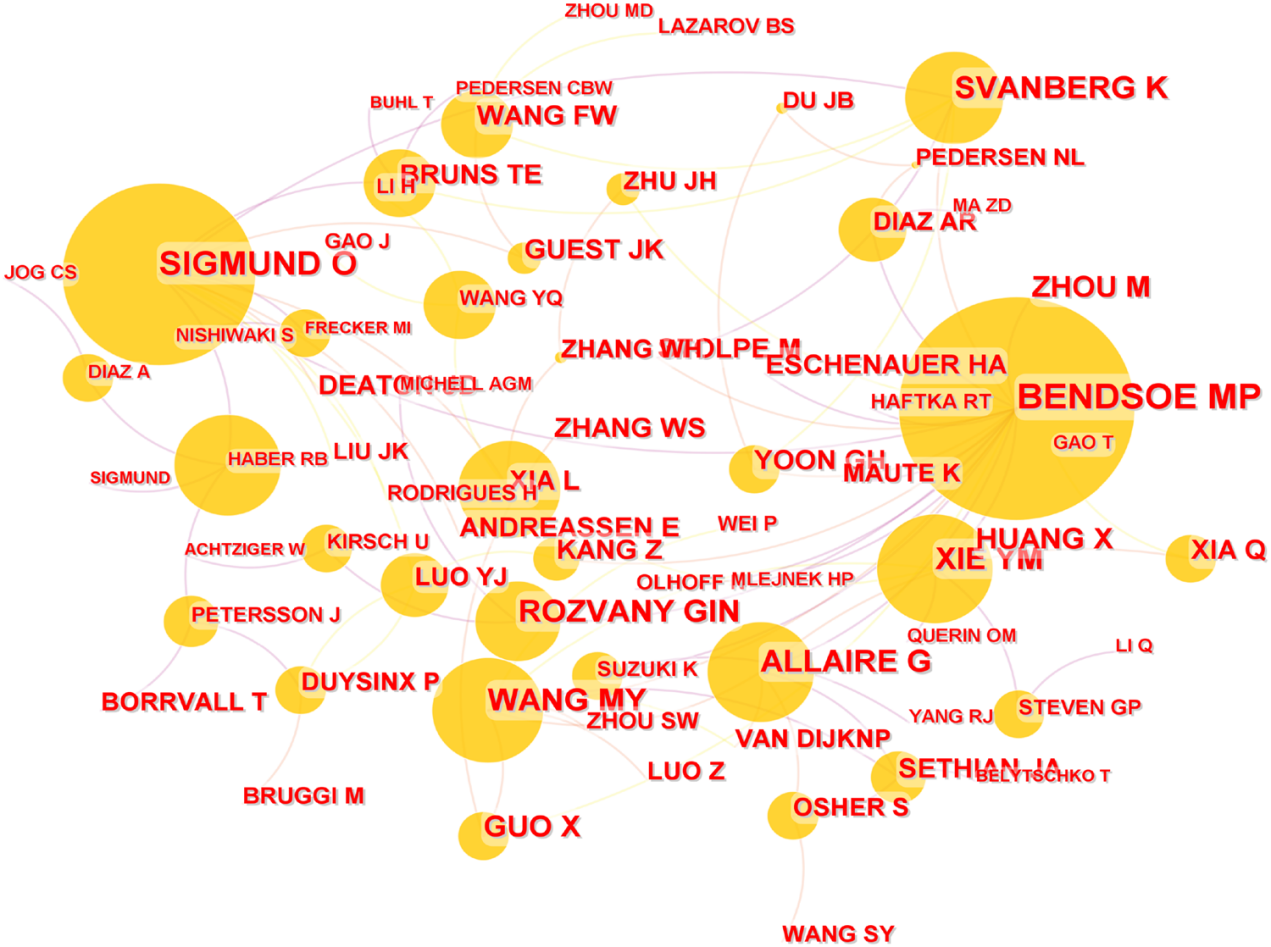
CS map of cited author analysis in the WOS literatures.

According to the analysis based on CiteSpace, the top ranked scholar by citation counts is M. Bendsøe with up to 3441 citation counts. The following scholars are O. Sigmund with 2741 citation counts, K. Svanberg with 1652 citations counts, G. Allaire with 1393 citation counts and G. Rozvany with 1273 citation counts. Top 10 scholars with high citation counts in the field of structural topology optimization in the WOS literatures is shown in Table 10. The CS map of cited author analysis in the WOS literatures is shown in Fig. 7.

**Table 10:** Top 10 scholars with high citation counts in the field of structural topology optimization in the WOS literatures.

Name of scholar	Count	Centrality
M. Bendsøe	3441	1.04
O. Sigmund	2741	0.68
K. Svanberg	1652	0.17
G. Allaire	1393	0.22
G. Rozvany	1273	0.13
M. Wang	1231	0.23
Y. Xie	1100	0.24
M. Zhou	947	0
X. Guo	834	0.05
X. Huang	676	0

In a certain sense, the higher the number of articles cited, it could indicate that the articles of these scholars have been recognized by other scholars in the field, and it has important reference significance for subsequent further research. Comparing Tables 8 and 10, there are some scholars mentioned in both tables, such as O. Sigmund, M. Wang, Y. Xie, X. Guo and X. Huang. This may could show that these scholars have contributed to the development of this field in terms of the number of publications and the number of citation counts.

### Co-authors’ institutions analysis

**Figure 8:**
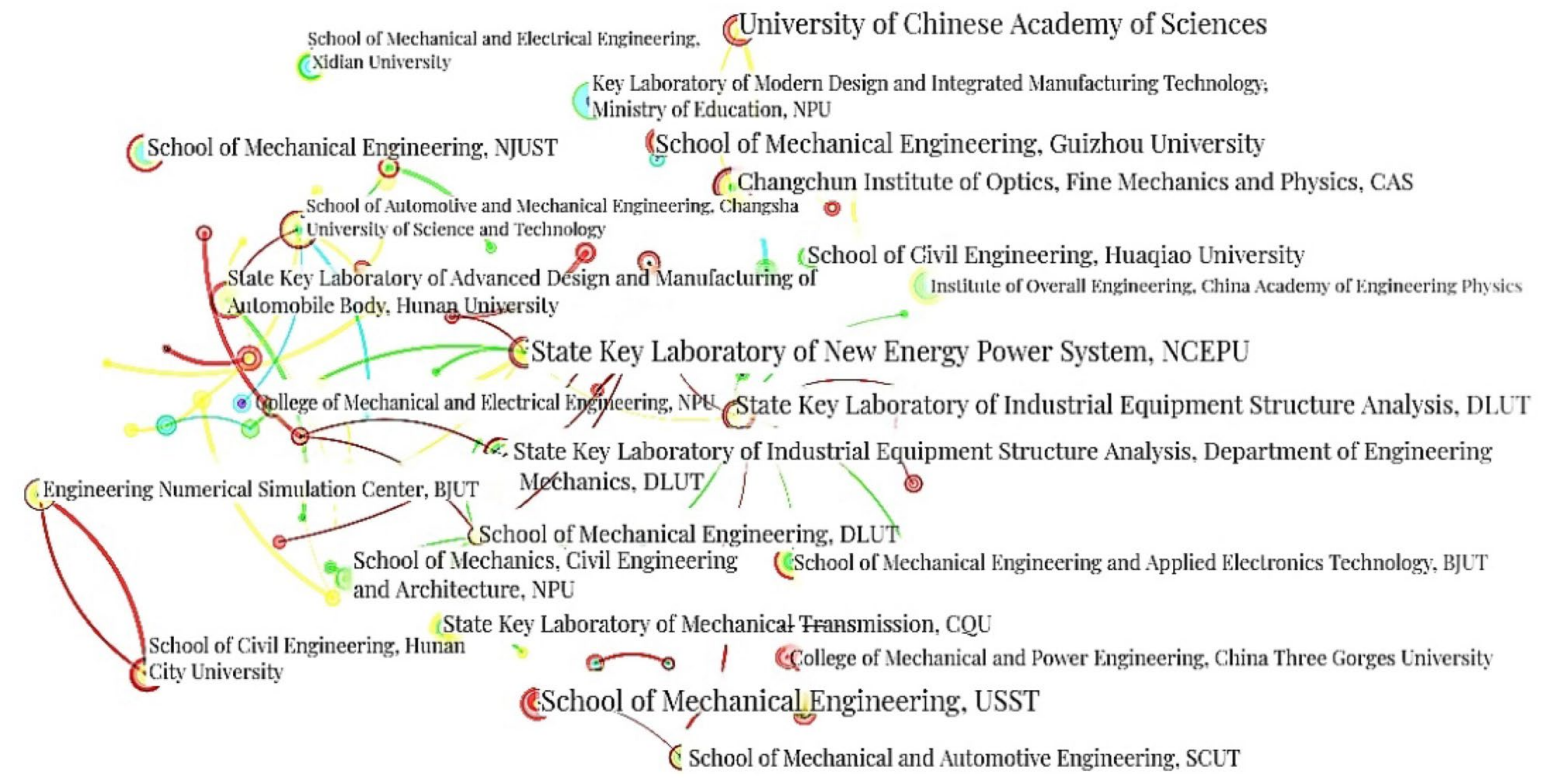
CS map of co-authors’ institutions analysis in the CNKI documents.

“Institution” is selected as the node type, and the top 50 data are chosen when analyzing the co-authors’ institutions in the CNKI documents. The CS map of institution co-authorship analysis comprises 286 nodes and 117 links, and the collaboration network density is 0.0029, which is shown in Fig. 8. The analysis parameters and result parameters are shown in Table 11.

**Table 11:** Analysis parameters and result parameters for co-authors’ institutions analysis in CNKI literatures.

	Parameters	Value
Analysis	Years per slice	4 years
	Node type	Institution
	Selection criteria	Top 50%
	Pruning	Pruning and pathfinder sliced network
Result	Nodes	286
	Links	117
	Density	0.0029

In Fig. 8, the nodes characterized with these five institutions (State Key Laboratory of Structural Analysis for Industrial Equipment, Dalian University of Technology^50^, Changchun Institute of Optics, Fine Mechanics and Physics, Chinese Academy of Science, Changchun^51^, School of Mechanical Engineering, University of Shanghai for Science and Technology^52^, University of Chinese Academy of Sciences^53^, and School of Automotive and Mechanical Engineering at Changsha University of Science and Technology^54^) are relatively bigger. This represents that these institutions have a larger quantity of publications. These institutions have strong academic research strength in the field of structural topology optimization.

Furthermore, more universities are shown in Fig. 8. This indicates that universities are the main force in the development of structural topology optimization at the current stage, and they have achieved fruitful achievements. These productive universities have a certain guiding role in the trend and direction of structural topology optimization research.

In terms of co-authors’ institutions analysis, the number of links is 117. No obvious clustering has been formed. It indicates that the cooperation between institutions is not close enough, and independent research is mainly conducted. The extent of institution co-authorship needs to be strengthened.

The main co-authors’ institutions in the CNKI documents include universities, enterprises and research institutes. Table 12 summarizes the three most prominent institutions in different categories in the CNKI documents arranged by the number of their original articles.

**Table 12:** Top 3 high publication co-authors’ institutions in three categories in the CNKI documents.

Category	Name of institution	Count
University	DUT	129
	NPU	104
	BJUT	87
Enterprise	Chang Guang Satellite Technology Co., Ltd	5
	DMTG	3
	Shanghai Aerospace Equipment Manufacturing Co., Ltd	2
Research institute	CIOMP	33
	CAEP	16
	CAST	4

The top three productive universities are Dalian University of Technology^50^, Northwestern Polytechnical University^55^, and Beijing University of Technology^56^. Chang Guang Satellite Technology Co., Ltd.^57^ ranks first among the enterprises, with 5 articles published followed by Dalian Machine Tool Group Co., Ltd. (3)^58^.The quantity of papers published by other enterprises is less than 3 articles each.

In research institutes, Changchun Institute of Optics, Fine Mechanics and Physics, Chinese Academy of Sciences^51^ reach 33 articles. Institute of Systems Engineering, China Academy of Engineering Physics^59^ and China Academy of Space Technology^60^ followed closely, with publication volumes of 16 and 4 articles, respectively.

Most of the research is completed by these institutions alone, and cooperation between institutions is relatively rare. Although there are certain research cooperation groups, they are limited to institutions with higher publication volumes.

In conclusion, in the CNKI documents, the number of articles published by universities is much higher than that of enterprises and research institutes. The number of publications by research institutes is also higher than that of enterprises.

**Figure 9:**
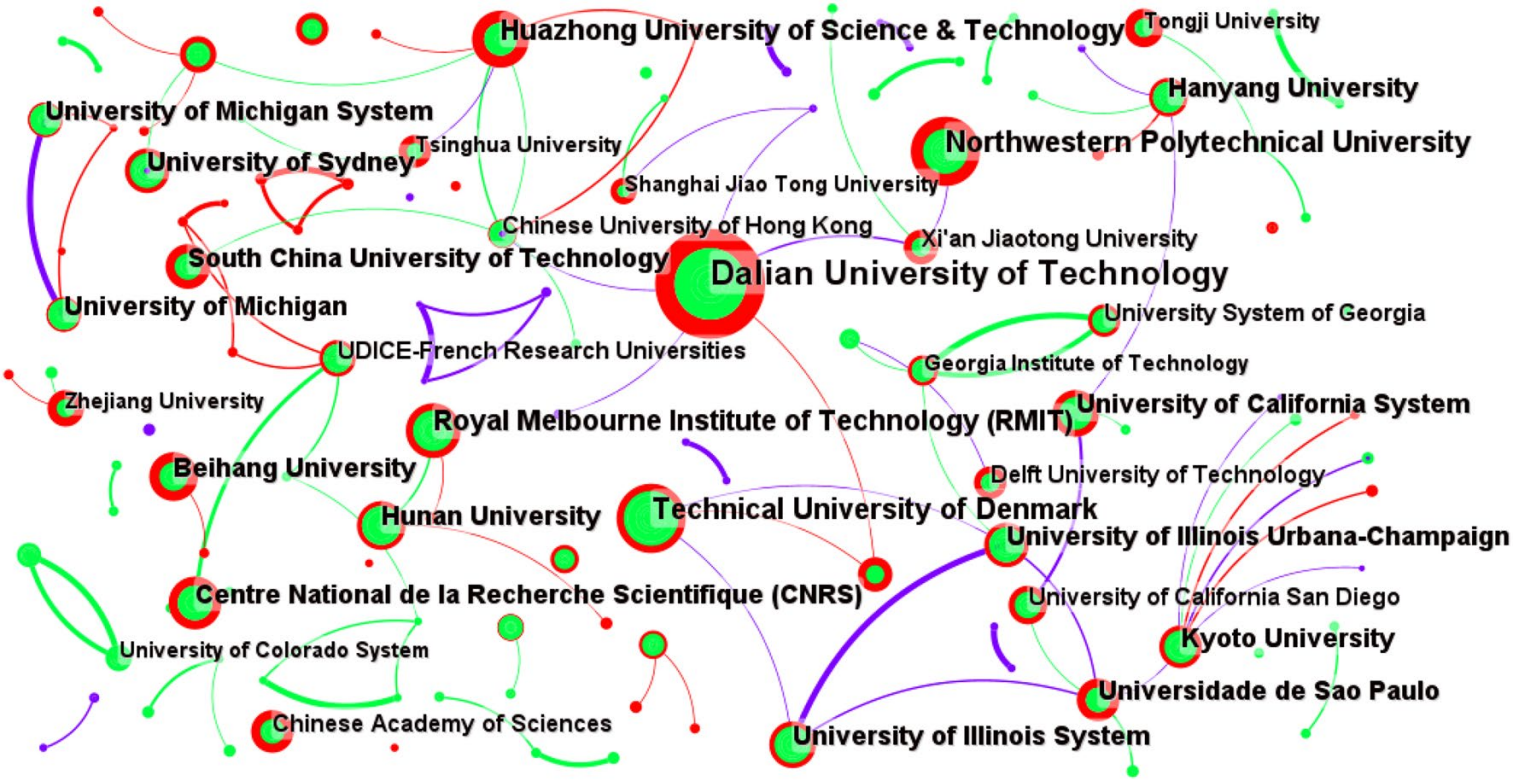
CS map of co-authors’ institutions analysis in the WOS documents.

The WOS documents are analyzed with a time slice of 11 years. “Institution” is selected as the node type, and the top 50 data are chosen. The CS map of co-authors’ institutions analysis in the WOS documents comprises 492 nodes and 729 links, with the collaboration network density of 0.006, which is shown in Fig. 9. The analysis parameters and result parameters are shown in Table 13.

**Table 13:** Analysis parameters and result parameters for co-authors’ institutions analysis in WOS documents.

	Parameters	Value
Analysis	Years per slice	11 years
	Node type	Institution
	Selection criteria	Top 50%
	Pruning	Pruning and pathfinder sliced network
Result	Nodes	492
	Links	729
	Density	0.006

As shown in Fig. 9, nodes labeled with universities such as Dalian University of Technology^12^, Technical University of Denmark^61^, Northwestern Polytechnical University^62^, and Huazhong University of Science & Technology^63^ are bigger. That is to say these co-authors’ institutions have published more papers in this field. Universities are the main contributors to the development of structural topology optimization. In terms of co-authors’ institutions analysis, there are 729 links. Obviously, some nodes are closely connected. It means these institutions form distinct research groups.

The main co-authors’ institutions in the WOS documents include universities, enterprises and research institutes. The three most prominent institutions in different categories in the WOS documents arranged by the number of their original articles is shown in Table 14.

On the one hand, the three universities with the most papers published are Dalian University of Technology (485), Technical University of Denmark (217), and Northwestern Polytechnical University (207). On the other hand, Toyota Motor Corporation^64^ have the most original papers among enterprises, with 24 articles published, followed by Honda Motor Company (11)^65^. The publication output of other enterprises is less than 10 articles each. Moreover, among research institutes, the Centre National de la Recherche Scientifique (CNRS)^66^ ranks the first, having 118 original articles, following by the Chinese Academy of Sciences (75)^67^ and the United States Department of Energy (39)^68^.

As shown in Table 14, in the WOS documents, universities have most publications in the field of structural topology optimization. Moreover, research institutes also have more original papers than enterprises.

**Table 14:** Top 3 high publication co-authors’ institutions in three categories in the WOS literatures.

Category	Name of institution	Count
University	Dalian University of Technology	485
	Technical University of Denmark	217
	Northwestern Polytechnical University	207
Enterprise	Toyota Motor Corporation	24
	Honda Motor Company	11
	Hyundai Kia Motors	10
Research institute	Centre National de la Recherche Scientifique(CNRS)	118
	Chinese Academy of Sciences	75
	United States Department of energy	39

According to the analysis based on CS, universities account for 90% in the top ten productive institutions in terms of the quantity of original papers. Dalian University of Technology is the most fruitful in the international institutions, which means it has greatly promoted the in-depth research and progress of this field. Meanwhile, research institutes account for 10%.

Overall, both in the CNKI and WOS, universities are the leaders in the field of structural topology optimization. Research institutes have also made contributions in this field, while enterprises have less attention and research depth in this field.

## Keywords of structural topology optimization analysis

"Keyword" is set as the clustering option and other parameter types is the same as the parameters in author analysis in CS. In the analysis of keyword centrality, frequency represents the number of times a keyword appears. Centrality represents the importance of the keyword. The higher the frequency of a keyword, the more it reflects the popularity of the research direction.

### Keywords co-occurrence and centrality analysis

**Figure 10:**
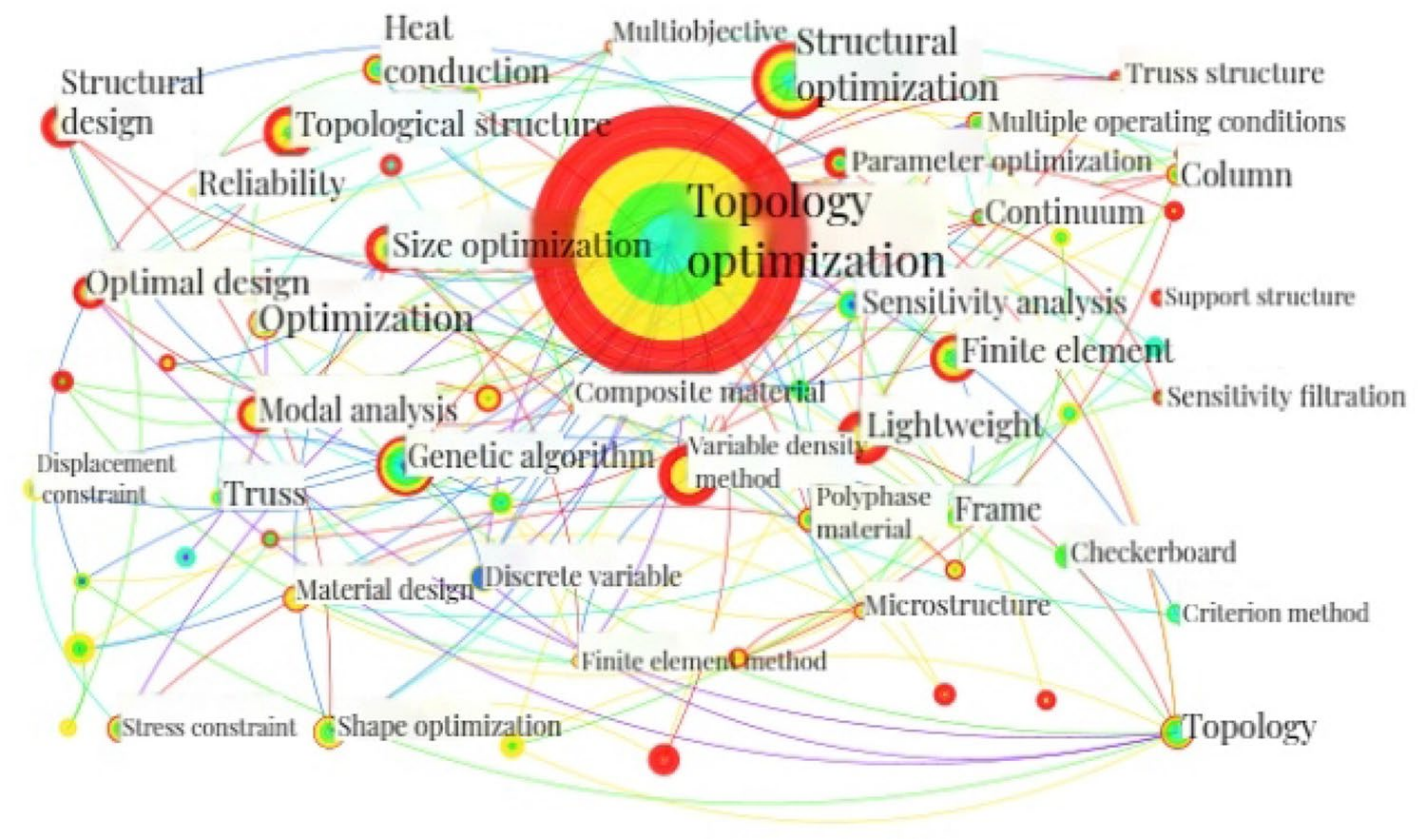
CS map of keywords analysis in the CNKI documents.

In CiteSpace, a time slice of 4 years is set to obtain a co-occurrence network map of CNKI documents keywords, which is shown in Fig. 10. The map comprises 406 nodes, 669 links, and a collaboration network density of 0.0081. The analysis parameters and result parameters are shown in Table 15.

**Table 15:** Analysis parameters and result parameters for keywords co-occurrence and centrality analysis in CNKI literatures.

	Parameters	Value
Analysis	Years per slice	4 years
	Node type	Keyword
	Selection criteria	Top 50%
	Pruning	Pruning and pathfinder sliced network
	Cluster algorithm	LLR
Result	Nodes	406
	Links	669
	Density	0.0081
	Modularity Q	0.6523
	Mean Silhouette	0.8854

A shown in Fig. 10, the research hotspots in the field of structural topology optimization in the CNKI documents include keywords such as "variable density method"^69^, "lightweight"^70^, "genetic algorithm"^71^, "size optimization"^72^, "finite element"^73^, "modal analysis"^74^, "stress constraint"^4^, "multiple loading cases"^75^, "truss structure"^76^, etc.

**Figure 11:**
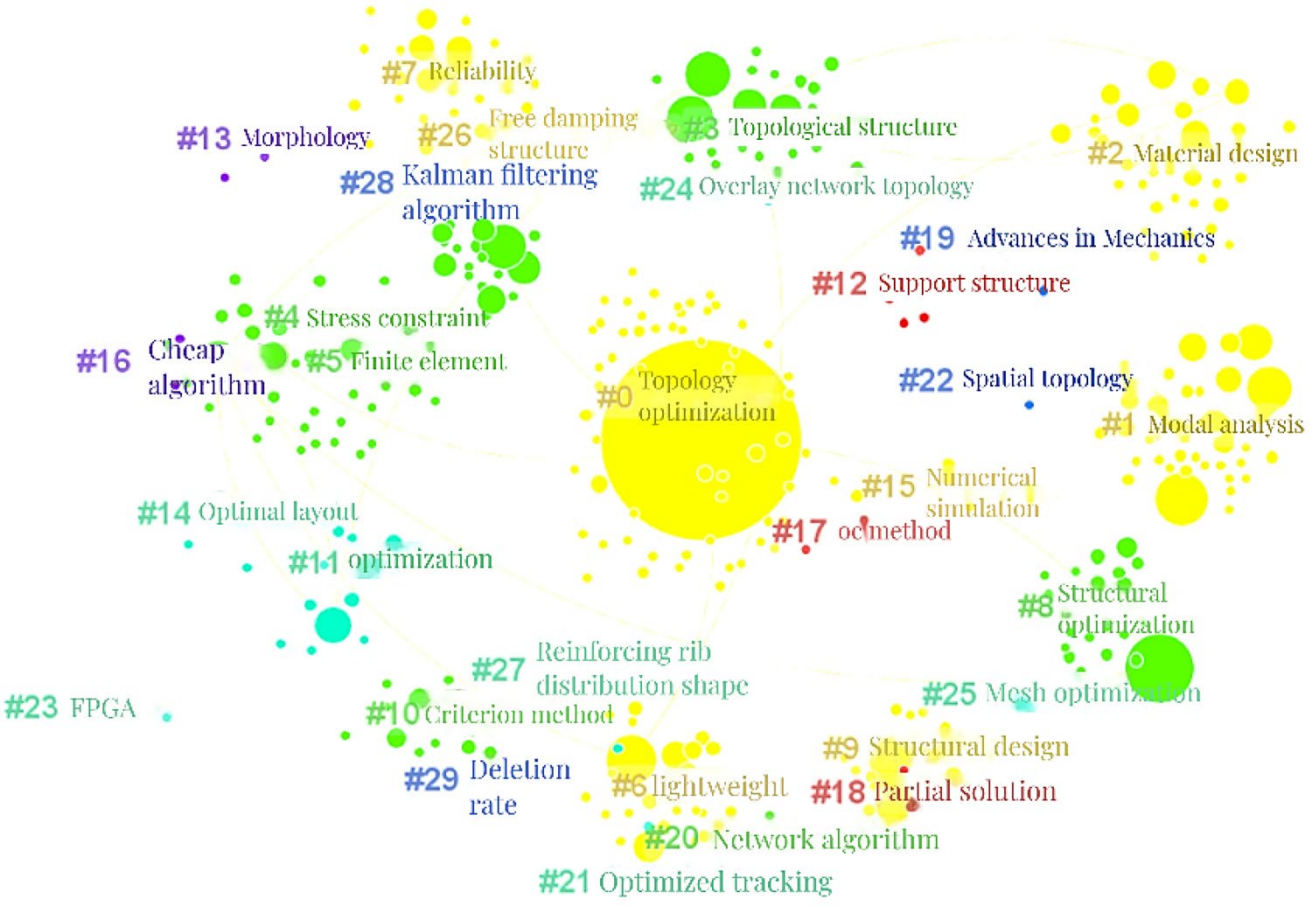
CS map of keyword cluster in the CNKI literatures.

Based on Fig. 10, the "Cluster" function is selected to obtain the keyword cluster CS map of CNKI documents with the Log Likelihood Ratio (LLR) algorithm, which is shown in Fig. 11. The value of Modularity Q is 0.6523 (greater than the critical value of 0.3), indicating a good cluster effect of the keyword network. The value of Mean Silhouette is 0.8854 (greater than the critical value of 0.5), indicating a reasonable cluster effect. The analysis parameters and result parameters are shown in Table 15.

"Summary Table" is selected and the top nine keyword is clustered to obtain the keyword clusters table as shown in Table 16.

**Table 16:** Top 9 keyword clusters in the CNKI literatures.

Cluster-ID	Size	Label(LLR)
0	66	Topology optimization, topology structure, strain energy, lightweight design, structure topology optimization
1	40	State analysis, size optimization, variable density method, column, sensitivity
2	35	Material design, negative Poisson's ratio, composite materials, microstructure, metamaterials
3	33	Topological structure, genetic algorithm, optimal design, topological optimization, truss structure
4	31	Structural topology optimization, stress constraint, sensitivity analysis, displacement constraint, steel frame structure
5	30	Finite element, truss, topology, parameter optimization, fatigue life
6	28	Lightweight, multiple operating conditions, crashworthiness, space camera, morphology optimization
7	27	Reliability, heat transfer, random variables, multiple materials, heat dissipation weakness
8	27	Structural optimization, filter function, solid mechanics, mathematical programming, checkerboard format

As shown in Fig. 11 and Table 16, the main research hotspots could be summarized into 11 clusters: "topology optimization (#0)"^77–79^, "modal analysis (#1)"^80–82^, "material design (#2)"^81,83^, "supporting structure (#3)"^84,85^, "stress constraint (#4)"^4,86^, "finite element (#5)"^87,88^, "lightweight design (#6)"^89,90^, "reliability (#7)"^91,92^, "structural optimization (#8)"^93,94^, "numerical simulation (#9)"^95,96^, and "criterion method (#10)"^97,98^.

The centrality analysis of the keywords in the CNKI documents is shown in Table 17. "Structural optimization"(0.17), "finite element"(0.16), and "topology"(0.15) have higher centrality, all exceeding 0.15. With the development of manufacturing processes, computer technology, and high-tech, keywords such as "additive manufacturing", "numerical simulation", and "deep learning" gradually became research hotspots after 2016, with centrality values of 0.08, 0.02, and 0.01, respectively.

**Table 17:** Centrality of keywords in the CNKI documents.

Keywords	Count	Centrality	Year
Topology	33	0.15	1999
Structural optimization	124	0.17	2003
Finite element	52	0.16	2004
Modal analysis	33	0.12	2007
Ansys workbench	11	0.01	2011
Shell structure	4	0.06	2012
AM	20	0.08	2016
Numerical simulation	2	0.02	2017
Deep learning	4	0.01	2021

**Figure 12:**
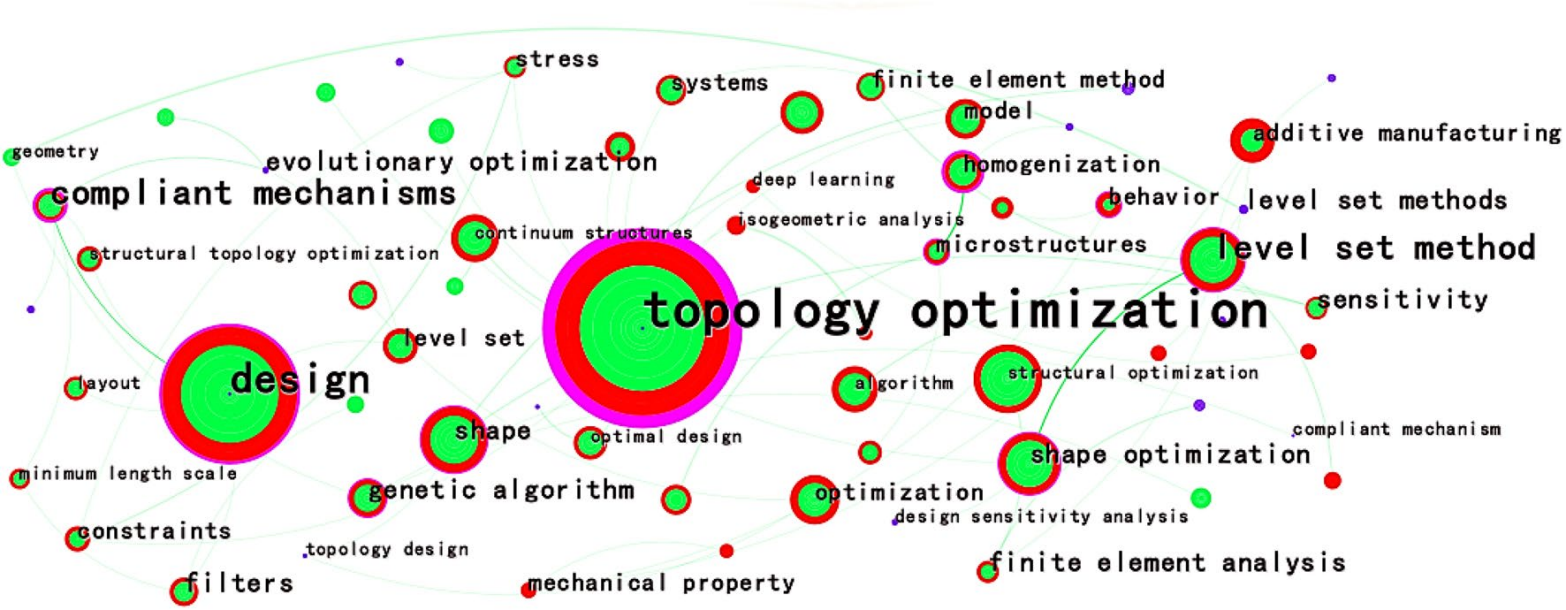
CS map of keywords co-occurrence in the WOS documents.

**Figure 13:**
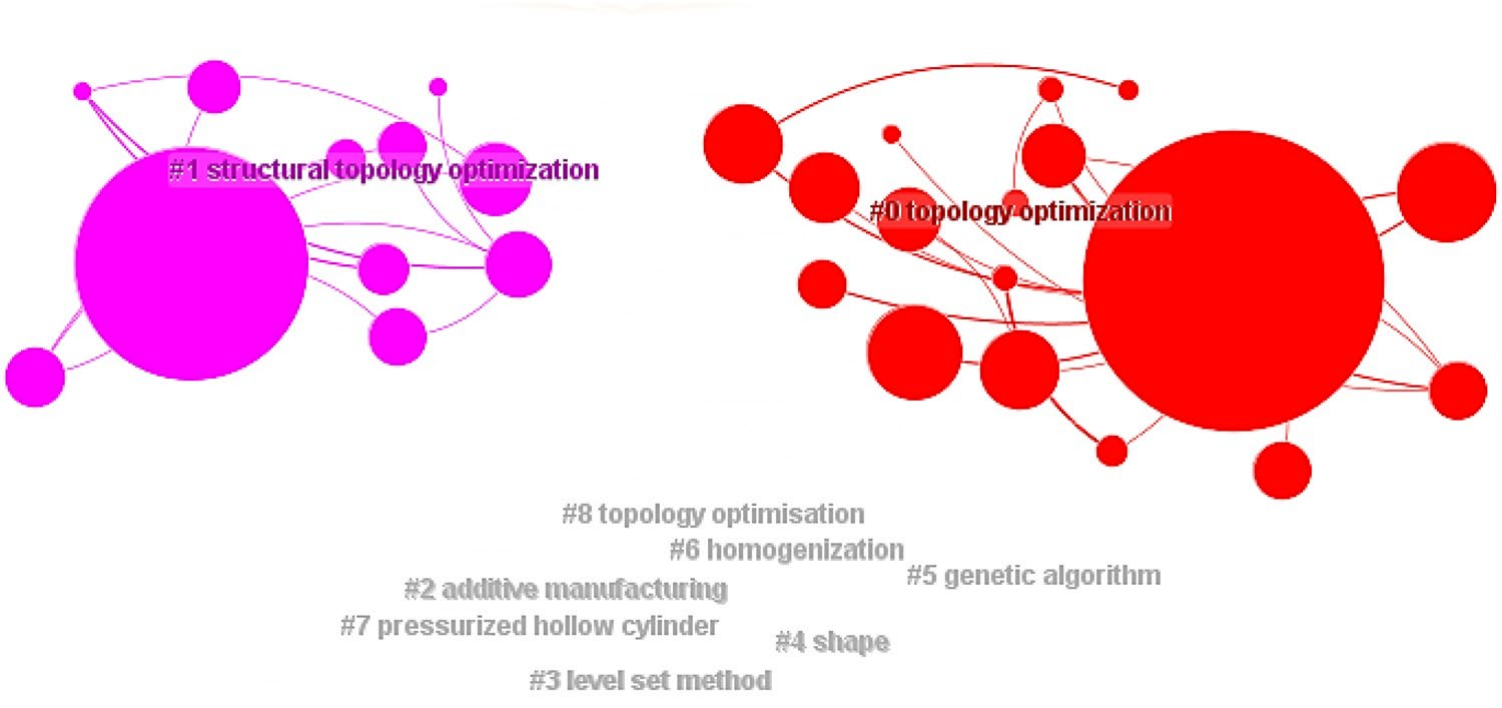
CS map of keyword clusters in the WOS documents.

In the analysis of the WOS documents, a time slice of 11 years is set with top 40 data to obtain a CS map of keywords co-occurrence, which is shown in Fig. 12. The map comprises 64 nodes, 86 links, and a collaboration network density of 0.0427. The value of Modularity Q is 0.5522 (greater than the critical value of 0.3), indicating a good cluster effect of the keyword network. The value of Mean Silhouette is 0.8243 (greater than the critical value of 0.5), indicating a reasonable cluster effect. The Log Likelihood Ratio (LLR) algorithm is selected to obtain the CS map of keyword clusters in WOS documents, which is shown in Fig. 13. The analysis parameters and result parameters are shown in Table 18.

**Table 18:** Analysis parameters and result parameters for keywords co-occurrence and centrality analysis in WOS literatures.

	Parameters	Value
Analysis	Years per slice	11 years
	Node type	Keyword
	Selection criteria	Top 40%
	Pruning	Pruning and pathfinder sliced network
	Cluster algorithm	LLR
Result	Nodes	68
	Links	86
	Density	0.0427
	Modularity Q	0.5522
	Mean silhouette	0.8243

As shown in Fig. 12, the research hotspots in the field of structural topology optimization in the WOS documents include keywords such as "topology optimization"^99^, "design"^100^, "structural optimization"^101^, "level set method"^102^, "continuum structures"^10^, "algorithm"^103^, "sensitivity analysis"^104^, "homogenization"^10^, and "genetic algorithm"^105^.

Based on Fig. 13, "Summary Table" in the "Cluster" menu bar is selected with the LLR algorithm. Then, the top five keyword clusters are chosen to obtain the top 5 keyword clusters, which is shown in Table 19.

**Table 19:** Top 5 keyword clusters in the WOS documents.

Cluster-ID	Size	Label(LLR)
0	17	Topology optimization, stress constraints, sensitivity analysis, additive manufacturing, optimal design
1	11	Structural topology optimization, compliant mechanisms, level set, genetic algorithms, minimum length scale
2	8	Additive manufacturing, finite element analysis, structural optimization, topology optimization, lattice structure
3	8	Level set method, shape optimization, finite element method, topological derivative, additive manufacturing
4	7	Shape, optimization, algorithm, topology design, stress constraints

Based on Figs. 12, 13, and Table 19, the main research hotpots of topology optimization in the WOS documents could be summarized into seven clusters: "topology optimization (#0)"^106,107^, "structural topology optimization (#1)"^108,109^, "additive manufacturing (#2)"^110,111^, "level set method (#3)"^102,112^, "shape (#4)"^113,114^, "genetic algorithm (#5)"^105,115^, and "homogenization (#6)"^116,117^.

Centrality analysis of keywords in the WOS documents is shown in Table 20.

**Table 20:** Centrality of keywords in the WOS documents.

Keyword	Count	Centrality	Year
Shape optimization	679	0.14	1999
Complicant mechanisms	190	0.26	1999
Level set method	556	0.2	2003
Microstructures	112	0.12	2010
Optimal design	265	0.06	1999
Additive manufacturing	256	0.06	2013
Isogeometric analysis	39	0.06	2021
Deep learning	26	0.06	2021

"Compliant mechanisms"(0.26) and "level set method"(0.2) have higher centrality, both exceeding 0.2. With the development of manufacturing processes, computer technology, and high-tech, keywords such as "additive manufacturing", "isogeometric analysis"^118^, and "deep learning" appeared in 2013 and 2021, and their centrality exceed 0.05, reaching 0.06.

In a conclusion, "topology", "structural optimization", and "shape optimization" are keywords with high frequency and centrality in both CNKI and WOS documents. They appeared earlier than other keywords, indicating that they were the research focus in the early stage. With the passage of time and the development of science and technology, keywords related to high-tech auxiliary technologies gradually emerged, such as "additive manufacturing", "numerical simulation", and "deep learning", which are common in both CNKI and WOS documents, reflecting the increasingly close integration of structural topology optimization and high-tech.

### Analysis of word with important bursts

**Figure 14:**
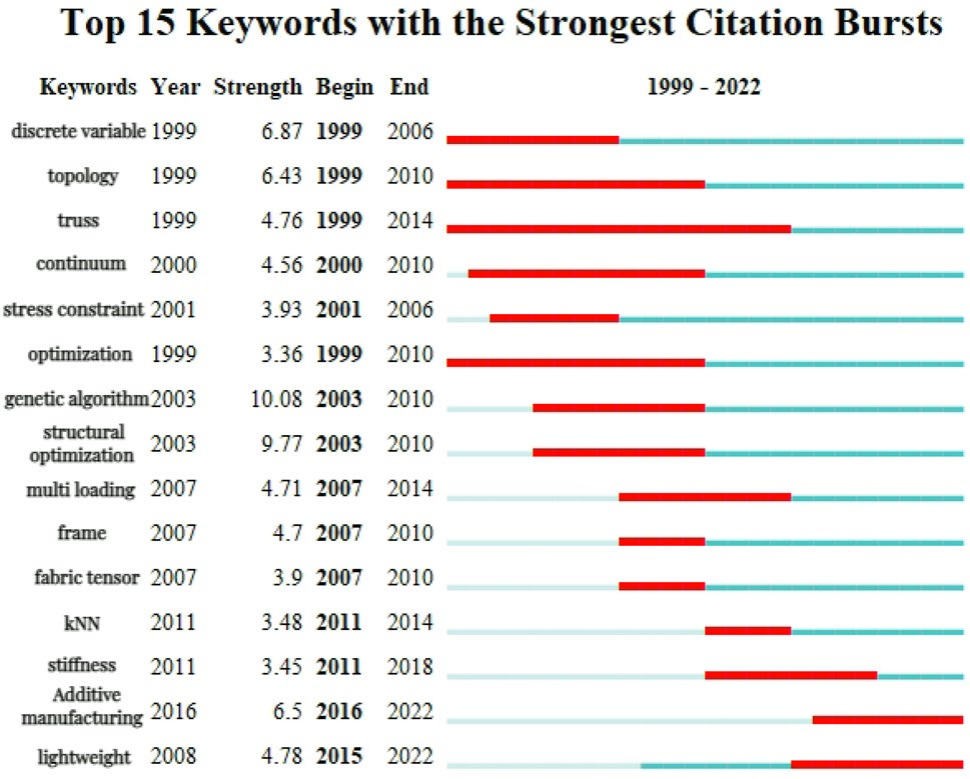
Top 15 Keywords with the strongest citation bursts in the CNKI documents.

**Figure 15:**
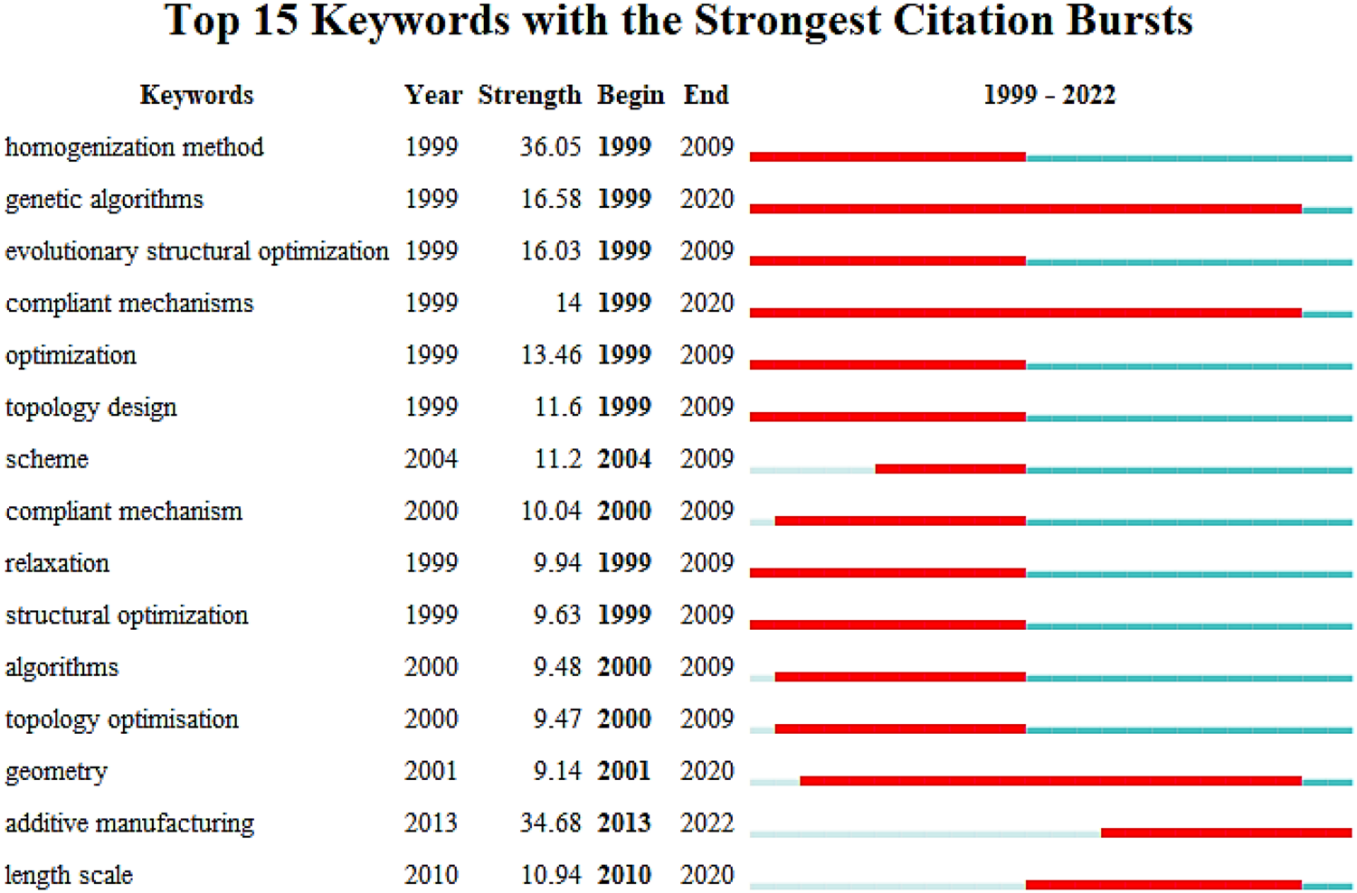
Top 15 keywords with the strongest citation bursts in the WOS documents.

Keeping up with the latest research frontiers could help understand the latest developments in the research field and focus on current hot issues. It could also help the overall understanding of the problems and shortcomings in the research, and lay a solid foundation for addressing deficiencies and further in-depth research. In CiteSpace, "Burstness" is selected as the basis of the keyword cluster map, the CS map of burst terms in the CNKI and WOS documents could be obtained, which is shown in Figs. 14 and 15.

The development of structural topology optimization over the past two decades could be divided into three periods.

The early period (1999–2010) is characterized by the burst terms such as “discrete variables”, “topology”, “truss”, “continuum”, “stress constraints”, and “genetic algorithms”.

The mid-period (2010–2014), is characterized by the bursts terms such as “structural optimization”, “k-nearest neighbors” (KNN), “multiple loading cases”, “fabric tensors”, and “MMC”.

The latest research frontiers, from 2011 to 2022, are characterized by keywords such as, “stiffness”, “additive manufacturing”, and “lightweight”.

### Analysis of algorithms for solving mathematical optimization problems

All kinds of structural topology optimization numerical methods require solving mathematical optimization problems finally. There are classical algorithms and heuristic algorithms to solve mathematical optimization problems.

Among them, the classical algorithms mainly include: criterion method and a series of sequence planning methods. Optimization methods based on heuristic algorithm can be divided into two categories: random search optimization method and evolutionary optimization method^34^.

Serial sequence planning methods and some criterion methods need to compute the derivatives (sensitivities) of the objective function and the constraint function. These methods generally have faster convergence speed and higher optimization efficiency, and are suitable for large-scale structural topology optimization. However, if the optimization problem itself is non-convex, derivatively-based methods may fall into local optimal solutions^119^.

The mathematical properties of optimization objective function and constraint function are seldom used heuristic algorithm, while heuristic algorithm can be easily combined with finite element analysis. The disadvantage of the algorithm is the lack of sufficient mathematical proof^34^.

It’s worth noting that due to the limitations of CiteSpace functions, this section analyzes classical algorithms and heurizing algorithms by using relevant keywords to search the CiteSpace filtered data mentioned in Sect. “Data sources and search strategy” for numerical statistics. Therefore, although the relevant analysis in this section is of reference significance, it still has limitations and incompleteness.

With “Optimality Criteria OR OC”, “Sequential Linear Programming OR SLP”, "Sequential Convex Programming OR SCP", "Sequential Quadratic Programming OR SQP" as keywords, relevant searches are conducted. 165 articles with key words including Optimality Criteria, 51 articles including Sequential Linear Programming, and 24 articles including Sequential Convex Programming and 48 articles on Sequential Quadratic Programming are obtained. In other words, in the database used in this paper, 288 articles can be obtained by this retrieval method.

With “Simulated Annealing Algorithm OR SAA”, “Genetic Algorithm OR GA”, "Tabu Search Algorithm OR TSA", "Evolutionary Structural Optimization OR ESO", “Bi-directional Evolutionary Structural OR BESO”, “Metamorphic Development OR MD” as keywords, relevant searches are conducted. 32 articles with key words including Simulated Annealing Algorithm, 662 articles including Genetic Algorithm, and 9 articles including Tabu Search Algorithm and 780 articles including Evolutionary Structural Optimization, 271 articles including Bi-directional Evolutionary Structural, 9 articles including MD are obtained. In other words, in the database used in this paper, 1763 articles can be obtained by this retrieval method. In view of incomplete statistical results, heuristic algorithm is more favored by researchers in the field of structural topology optimization.

## Highly co-cited documents analysis

In this part, the highly co-cited papers related to structural topology optimization are analyzed^120,121^. The top 5 most co-cited papers in the CNKI documents with citation count higher than 290 are shown in Table 21. Among the highly co-cited papers in the CNKI documents, the article by K. Zhou is co-cited most, reaching 679 times.

**Table 21:** Top 5 most co-cited CNKI documents on structural topology optimization.

Name of document	Author	Year	Co-cited count
A review on topology optimization of structures	K. Zhou	2005	679
Topological optimization design for continuum structures	Z. Luo	2004	464
An overview on the topological optimization design of structures	Z. Guo	2007	436
Research on multi-object topology optimization on bus chassis frame	W. Fan	2008	367
Topology optimization of continuum structure with stress and displacement constraints under multiple loading cases	Y. Sui	2000	297

K. Zhou et al.^122^ introduced the Michell theory in the analytical method of structural topology optimization. The numerical methods for topology optimization of truss and continuum structures are highlighted in the paper. When analyzing the truss structure, the ground structure method is often used. However, when analyzing the continuum structure, the topology optimization is often achieved by dividing the continuum structure into finite elements, and then deleting elements to forming perforated continuum. Moreover, the mechanism of numerical computation instability in the topology optimization process of continuum structures is also mentioned. Z. Luo et al.^123^ reviewed the topology optimization techniques for continuum structures based on finite element method through variety methods. On the one hand, density-stiffness interpolation format and optimization criteria method are used. On the other hand, a convex programming method based on mathematical programming, method of moving asymptotes (MMA) and density-based method. Moreover, the optimization design problems of multi-objective, flexible structures, and multi-physics field topology are discussed. The numerical calculation problems such as checkerboard pattern and grid dependence in the optimized structure are analyzed. The process flow of topology optimization design of continuum structures is provided. The effectiveness of the above methods is demonstrated by relevant algorithms. Z. Guo et al.^124^ reviewed the development and research status of structural topology optimization from two types of objects, including discrete and continuum. Based on the SIMP method, W. Fan et al.^125^ defined a multi-objective topology optimization method and multi-stiffness topology optimization function through the compromise programming approach. They also defined the objective function for vibration natural frequency topology optimization using the average frequency method. This research method is a multi-objective topology optimization research method suitable for continuum structures. Y. Sui et al.^126^ applied the topology optimization method of independent continuous mapping (ICM) for topological variables to continuum structures. A unified topology optimization model for continuum structures with weight as the objective, considering stress and displacement constraints, was also established. By comprehensively coordinating the displacement-stress topology solution, the coordinated topology solution was inverted from discrete to continuous according to the threshold. Then the problem named ill loading could be overcome by a multiple-level strategy and a weight factor.

Overall, the highly co-cited CNKI documents mainly focuses on theoretical research and method reviews, emphasizing the exploration of topology optimization methods for continuum structures. From multiple perspectives, such as the different characteristics of the structures, the weaknesses and difficulties of classical research methods, innovative breakthroughs and discussions are made.

The top 6 most co-cited articles with citation counts exceeding 150 in the WOS documents are shown in Table 22. The article by Deaton JD is co-cited most, reaching 261 times.

**Table 22:** Top 6 most co-cited WOS documents on structural topology optimization.

Name of document	Author	Year	Co-cited count
A survey of structural and multidisciplinary continuum topology optimization: post 2000	J. Deaton	2014	261
Topology optimization : theory, methods and applications	M. Bendsøe	2004	243
Topology optimization approaches A comparative review	O. Sigmund	2013	225
Topology optimization in aircraft and aerospace structures design	J. Zhu	2016	177
A new topology optimization approach based on Moving Morphable Components (MMC) and the ersatz material mode	W. Zhang	2016	167
Current and future trends in topology optimization for additive manufacturing	J. Liu	2018	155

J. Deaton et al.^127^ reviewed the progress of topology optimization for continuum structures from 2000 to 2012 in their article, including the SIMP method, the evolutionary structural optimization (ESO) method, level set method, and a new biologically inspired method based on cellular division rules. The review covered mature classical methods, expanded field ranges, and the introduction of new methods. M. Bendsøe et al.^128^ derived the topology design of isotropic materials by mathematical forms and introduced relevant application scenarios. Moreover, the article emphasized the importance of flexibility optimization and the use of composite materials in large-scale structural design. Relevant discussions are illustrated by the examples of truss. O. Sigmund et al.^129^ analyzed the advantages and disadvantages, similarities and differences of several related concepts such as "density level set", "topological derivative", "phase field" and "evolution" in the field of structural topology optimization. J. Zhu et al.^130^ summarized the current applications of topology optimization technology in aircraft and aerospace structural design in the article. Meanwhile, the potential application of topology optimization are introduced, including non-dynamic response design, shape-preserving design, smart structure design, structural feature design, and additive manufacturing. W. Zhang et al.^13^ proposed a topology optimization method based on the MMC solution framework, which is suitable for parts with variable thickness and greatly improves the efficiency of numerical solutions. This is achieved by properly constructing the topological description function of the components and utilizing ersatz material model through projecting the topological description function of the components. In their article, J. Liu et al.^131^ summarized the latest state-of-art topology optimization methods for a variety of additive manufacturing (AM) and pointed out the development trends and prospects of the current issues related to the diversity and complexity of AM processes.

In a conclusion, highly cited WOS documents mainly cover classic method reviews, mathematical derivations, industrial applications, and development trends, providing a more comprehensive theoretical methodology for the field of structural topology optimization. The content of the documents overlaps more widely and is closely related to each other.

## Summary

Based on CiteSpace, this paper statistically analyzes CNKI and WOS documents in the field of structural topology optimization over the past 20 years. The main content includes the following four parts: annual quantity of papers and productive countries, core authors and institutions, hotspots and burst terms, and the highly co-cited papers. The research results show that:

1. The number of papers published in the field of structural topology optimization has shown a fluctuating growth trend year by year. Under the same search strategy, there are less CNKI literature to be obtained than WOS literature. By statistical analysis, in the WOS literature, China ranks the first in the terms of the number of published documents, with a proportion of 34.30%, followed by the United States accounted for 18.52%, and Australia accounted for 6.72%.
2. Top 5 application areas of structural topology optimization in WOS literatures are Mechanics with 2827 articles searched, Engineering and Multidisciplinary with 2805 articles searched, Computer science and Interdisciplinary applications with 2131 articles, Mathematics and Interdisciplinary applications with 822 articles, Engineering and Mechanical with 765 articles.
3. The author with high publications in the field of structural topology optimization in the CNKI documents is Y. Sui with 58 published papers. Z. Kang and O. Sigmund rank the first in the WOS documents in the terms of the amounts of publications. They both published 86 articles.
4. In the aspect of cited authors, M. Bendsøe ranks first in the top 10 scholars with high citation counts in the field of structural topology optimization in the WOS literatures, with 3441 citation counts. O. Sigmund ranks second with 2741 citation counts, followed by K. Svanberg with 1652 citations.
5. Meanwhile, according to the analysis based on CS, universities rank the first in Top ten co-authors’ institutions with the amounts of published papers, with a proportion of about 90%. This analytical result indicates that universities have more in-depth research on structural topology optimization than enterprises and research institutes. Moreover, Dalian University of Technology has published the most papers both in the CNKI and WOS documents, with 129 articles and 485 articles respectively.
6. The research hotspots in the field of structural topology optimization focus on the keywords including: "level set method", "sensitivity analysis", "homogenization", "genetic algorithm", "variable density method", "finite element", "stress constraint", and "multiple loading cases", etc. With the development of processing and computer technology, keywords with the strongest citation bursts have become the latest hotspots, including “genetic algorithms”, “MMC”, “AM” and “deep learning”, etc. Currently, the hotspots in the field of structural topology optimization focus on the lightweight technology research, topology optimization design for three-dimensional engineering structures, methods for adding ersatz material models, and topology optimization design for additive manufacturing.
7. On the one hand, highly co-cited CNKI documents on structure topology optimization mainly focus on theoretical research and method reviews. Systematic comparisons and stage summaries of different research methods are obtained in these papers. On the other hand, highly co-cited WOS documents on this topic mainly includes digital derivation of theoretical methods and engineering applications. Moreover, the advantages and disadvantages of theoretical applications are analyzed through combining practical engineering cases in these papers.

With the development of high-tech and advanced manufacturing technologies, the computational efficiency and accuracy of structural topology optimization will be greatly improved in the future. This will provide stronger support for both theoretical research and industrial applications. The future research directions could be explored from the following four aspects:

### Multi-scale structural topology optimization

Structural topology optimization could evolve from macroscopic structural optimization to micro-topology optimization, achieving more precise structural design.

### Integration of structural topology optimization and additive manufacturing (AM)

Structural topology optimization usually considers structural performance as the first consideration, which increases the difficulty of manufacturing to some extent. In the future, research in this field would combine AM technology to manufacture more complex structures and further improve the structural performance.

### Integration of structural topology optimization and material design

Structural topology optimization usually only considers the shape of the structure, ignoring the influence of materials. Subsequent research directions would focus on combining structural topology optimization with material design to achieve more optimized structural design.

### Integration of structural topology optimization and artificial intelligence (AI)

By combining structural topology optimization with AI, more efficient and accurate structural optimization could be achieved through machine learning and other technologies.

In an era that places great emphasis on industrial efficiency, accelerating the research process of structural topology optimization technology is of great significance for achieving sustainable development and industrial upgrading.

## Data Availability

The data that support the findings of this study are available on request from the corresponding author.
